# Fat Metaplasia on Sacroiliac Joint Magnetic Resonance Imaging at Baseline Is Associated with Spinal Radiographic Progression in Patients with Axial Spondyloarthritis

**DOI:** 10.1371/journal.pone.0135206

**Published:** 2015-08-13

**Authors:** Kwi Young Kang, In Je Kim, Min A Yoon, Yeon Sik Hong, Sung-Hwan Park, Ji Hyeon Ju

**Affiliations:** 1 Division of Rheumatology, Department of Internal Medicine, Medical College, The Catholic University of Korea, Seoul, South Korea; 2 Division of Rheumatology, Department of Internal Medicine, Incheon St. Mary's Hospital, The Catholic University of Korea, Incheon, South Korea; 3 Division of Rheumatology, Department of Internal Medicine, College of Medicine, Ewha Womans University, Seoul, South Korea; 4 Department of Radiology, Korea University Guro Hospital, College of Medicine, Korea University, Seoul, South Korea; Toronto Western Hospital, CANADA

## Abstract

**Objective:**

To study the relationship between inflammatory and structural lesions in the sacroiliac joints (SIJs) on MRI and spinal progression observed on conventional radiographs in patients with axial spondyloarthritis (axSpA).

**Methods:**

One hundred and ten patients who fulfilled the ASAS axSpA criteria were enrolled. All underwent SIJ MRI at baseline and lumbar spine radiographs at baseline and after 2 years. Inflammatory and structural lesions on SIJ MRI were scored using the SPondyloArthritis Research Consortium of Canada (SPARCC) method. Spinal radiographs were scored using the Stoke AS Spinal Score (SASSS). Multivariate logistic regression analysis was performed to identify predictors of spinal progression.

**Results:**

Among the 110 patients, 25 (23%) showed significant radiographic progression (change of SASSS≥2) over 2 years. There was no change in the SASSS over 2 years according to the type of inflammatory lesion. Patients with fat metaplasia or ankyloses on baseline MRI showed a significantly higher SASSS at 2 years than those without (p<0.001). According to univariate logistic regression analysis, age at diagnosis, HLA-B27 positivity, the presence of fat metaplasia, erosion, and ankyloses on SIJ MRI, increased baseline CRP levels, and the presence of syndesmophytes at baseline were associated with spinal progression over 2 years. Multivariate analysis identified syndesmophytes and severe fat metaplasia on baseline SIJ MRI as predictive of spinal radiographic progression (OR, 14.74 and 5.66, respectively).

**Conclusion:**

Inflammatory lesions in the SIJs on baseline MRI were not associated with spinal radiographic progression. However, fat metaplasia at baseline was significantly associated with spinal progression after 2 years.

## Introduction

Axial spondyloarthritis (axSpA) is a chronic inflammatory disease that mainly affects the spine and the sacroiliac joints (SIJs). AxSpA falls into two categories: non-radiographic axSpA, in which there is no evidence of sacroiliitis on conventional radiographs, and ankylosing spondylitis (AS), in which there is definitive evidence of sacroiliitis [1]. Radiographic progression in the spine is strongly associated with spinal mobility and functional status, and therefore represents a clinically important outcome and treatment target in those with axSpA [2].

Spinal progression varies widely among axSpA patients. Previous studies have examined factors that influence the heterogeneous development of syndesmophytes in these patients. The strongest predictor of radiographic spinal progression is the presence of syndesmophytes at baseline [3,4]. In addition, increased levels of acute phase reactants and smoking are independent predictors of radiographic spinal progression in early axSpA patients [4].

The use of magnetic resonance imaging (MRI) as a tool for diagnosing axSpA is increasing. MRI can detect active inflammatory lesions in the SIJs, particularly on fat-suppressed (FS) images [5]. Furthermore, MRI can detect both post-inflammatory changes including MRI-specific fatty lesions (fat metaplasia) and chronic changes (sclerosis, erosion, and ankyloses), although the latter can also be detected by other imaging methods [2]. Fat metaplasia is an early post-inflammatory change [6], and most likely reflects the early stages of bone remodeling [7].

The advent of MRI has allowed noninvasive evaluation of the association between inflammatory/chronic lesions and new bone formation in axSpA. It is unclear whether active inflammation as detected by MRI in the spine is predictive of new bone formation [8–10]; however, fat metaplasia on spinal MRI does appear to predict the formation of new syndesmophytes [7,11], which are a potential starting point for new bone formation in AS patients [7]. Interestingly, the majority of new syndesmophytes (>50%) had no corresponding detectable spinal MRI lesions at baseline; this suggests that new bone formation in the spine may have a general systemic effect, rather than inducing local inflammation [11].

Although spinal MRI is useful for predicting spinal progression, it not required for a diagnosis of axSpA. However, MRI of the SIJs is an important practical tool for evaluating patients suspected of having early SpA [12]. Furthermore, evidence of SIJ inflammation on MRI correlates with disease activity and with the levels of systemic inflammatory markers such as C-reactive protein [13,14]. That said, no study has examined the utility of inflammatory lesions on SIJ MRI for predicting spinal radiographic progression. Also, it is not known whether post-inflammatory changes in the SIJs are associated with progression of spinal damage.

Therefore, the aim of the present study was to examine the association between SIJ findings on baseline MRI and radiographic spinal progression in patients with axSpA, and to identify predictors of spinal structural damage.

## Methods

This study enrolled 110 patients (83 men and 27 women) with axSpA who fulfilled the Assessment of SpondyloArthritis international Society (ASAS) axSpA criteria [12] and who were followed up at Incheon Saint Mary’s hospital. All 110 patients underwent baseline MRI scans of the SIJs. Radiographs of the lumbar spine were obtained at the time of MRI and after 2 years.

Demographic data included age, gender, age at the time of axSpA diagnosis, disease duration, a history of uveitis, peripheral arthritis, enthesitis, and a family history of axSpA. Inflammatory markers (C-reactive protein (CRP) and the erythrocyte sedimentation rate (ESR)) were measured at the time of MRI examination. Medications, including non-steroidal anti-inflammatory drugs (NSAIDs), sulfasalazine, methotrexate and tumor necrosis factor (TNF) inhibitors, were recorded. The participants’ written consent was obtained according to the Declaration of Helsinki. The study was approved by the ethics committee at Incheon Saint Mary’s hospital.

### Radiographs and scoring

To obtain the SASSS, the anterior and posterior vertebrae of the lumbar (T12 lower to S1 upper) spinal segments were scored on a scale of 0 to 3 as follows: 0 = normal, 1 = erosion, sclerosis, or squaring; 2 = syndesmophyte formation; and 3 = a bridging syndesmophyte [15]. A change in the SASSS of ≥2 in 2 years was defined as significant; such patients were allocated to the SASSS progression group. The interclass correlation coefficient (ICC) for baseline scores and change scores (scores that changed from baseline over the 2 year period) were calculated to quantify the reliability of radiographic scoring. To calculate this, two readers (both of whom were blinded to the sequence of the films) scored 50 paired radiographs (at baseline and after 2 years). The ICC for the baseline scores was 0.83 and that of change scores was 0.71. An additional analysis was performed using scores assigned by a single reader.

### MRI protocol

MRI of the SIJs was performed at baseline. Images were obtained using a 3.0 T MRI unit (Verio/Skyra; Siemens Medical, Erlangen, Germany) and a body flexed array coil (Siemens Medical, Erlangen, Germany). Assessment of structural lesions in the SIJs was based on T1-weighted turbo spin echo (TSE) MRI sequences. Assessment of inflammatory lesions was based on T2-weighted FS TSE sequences. The sequence protocols were as follows: semi-coronal (along the long axis of the sacral bone) TSE (slice thickness (ST) 3 mm; repetition time/echo time (TR/TE) 636/11 ms), and semi-coronal T2-weighted FS TSE (ST 3 mm; TR/TE 5210/55 ms).

### Semi-quantitative assessment of MRI findings

Inflammatory and structural lesions on SIJ MRI were scored according to the SPondyloArthritis Research Consortium of Canada (SPARCC) method [16,17]. All scores were measured by two experienced investigators (one musculoskeletal radiologist and one rheumatologist), who had completed the SPARCC online MRI training module.

Assessment of inflammatory lesions in the SIJs was based on T2-weighted FS TSE images. The SIJs were scored using the SPARCC method [16] based on six consecutive coronal slices depicting the synovial portion of the joint. The scoring ranges were as follows: bone marrow edema, 0–48; depth, 0–12; and intensity, 0–12. The maximum score is 72.

Structural lesions on SIJ MRI were scored according to standardized definitions using a semi-quantitative scoring method, the SPARCC SI structural lesion score (SSS) [17]. The SSS was based on T1-weighted TSE images, with slices selected according to well-defined anatomical principles. Scoring was dichotomous (lesion present/absent) and based on five consecutive slices through the cartilaginous portion of the joint. The scoring ranges were as follows: fat metaplasia, 0–40; erosion, 0–40; backfill, 0–20; and ankylosis, 0–20. The maximum score is 120.

### Statistical analysis

Statistical analyses were performed using PASW statistics 18 (SPSS Inc., Chicago, IL, USA). Continuous data were expressed as the mean ± SD and categorical data as percentages. Normally distributed variables were compared using an independent t-test and non-normally distributed variables were compared using the Mann-Whitney U test. The Chi-squared test was used to compare categorical variables. Spearman’s correlation coefficient was used to analyze the correlation between variables.

The interobserver reliability was calculated using analysis of variance to yield an ICC. A two-way random model for absolute agreement was used. To reduce the effects of interobserver variation, an additional analysis was performed using scores measured by one radiologist. Changes in the SASSS according to the severity of fat metaplasia were estimated using analysis of covariance (ANCOVA) after adjusting for confounders. For ANCOVA, we selected covariates using the Akaike information criterion. The selected covariates were SASSS at baseline and TNF inhibitor treatment.

Multivariate logistic regression analysis was performed to investigate the utility of MRI findings for predicting spinal progression after adjusting for potential confounders. All variables with a p value < 0.10 in the univariate analysis were incorporated as explanatory variables. Variables with p value < 0.05 were entered into multivariate stepwise regression analyses (forward selection) whereas those with a p value > 0.1 were eliminated. A p value < 0.05 was considered statistically significant. The accuracy of the multivariate models was measured by the area under the curve (AUC).

## Results

The baseline characteristics of the 110 axSpA patients are listed in Table 1. The mean age of the patients was 32±11 years, and 83 (75.5%) were male. The average age at the time of axSpA diagnosis and mean disease duration were 28±10 and 3±5 years, respectively. Ninety-seven patients (88%) were HLA-B27 positive and 47 (43%) were taking TNF inhibitors. The baseline SASSS was 5.3±9.1. Twenty-nine patients (26%) had one or more syndesmophytes at baseline.

**Table 1:** Baseline characteristics of patients with axial spondyloarthritis.

Parameter at baseline		Radiographic progression		
	Total (n = 110)	Absent (n = 85)	Present (n = 25)	p value
Male (%)	83 (75.5)	62 (72.9)	21 (84.0)	0.303
Age (years)	31.6±10.6	29.3±9.8	39.6±9.5	<0.001
Age at diagnosis (years)	28±10	26±10	36±10	<0.001
Duration since diagnosis (years)	3.0±5.0	2.9±5.1	3.2±5.0	0.756
HLA-B27 positive (%)	97 (88.2)	78 (91.8)	19 (76)	0.070
Peripheral arthritis (%)	63 (57.3)	49 (57.6)	14 (56.0)	1.000
Enthesitis (%)	29 (26.4)	24 (28.2)	5 (20.0)	0.606
Uveitis (%)	28 (25.5)	21 (24.7)	7 (28.0)	0.796
Family history (%)	13 (11.8)	11 (12.9)	2 (8)	0.729
ESR (mm/hr)	24.6±23.5	24.2±23.3	26.±24.4	0.443
CRP (mg/l)	11.8±19.5	10.5±19.5	16.2±19.1	0.051
NSAIDs (%)	100 (90.9)	78 (91.8)	22 (88.0)	0.692
Sulfasalazine (%)	36 (32.7)	32 (37.6)	4 (16.0)	0.053
Methotrexate (%)	38 (34.5)	26 (68.2)	12 (48.0)	0.150
TNF inhibitors (%)	47 (42.7)	32 (37.6)	15 (60.0)	0.065
SASSS	5.3±9.1	2.5±5.8	4.2±3.6	<0.001
SASSS ≥2 (%)	50 (45.5)	29 (34.1)	21 (84.0)	<0.001
Number of Syndesmophytes	1.35±2.82	0.5±1.9	4.2±3.6	<0.001
Presence of syndesmophytes (%)	29 (26.4)	11 (12.9)	18 (72.0)	<0.001

ESR, erythrocyte sedimentation rate; CRP, C-reactive protein; NSAIDs, Non-steroidal anti-inflammatory drugs; TNF, tumor necrosis factor; SASSS, Stokes ankylosing spondylitis spine score

Twenty-five patients (23%) showed significant radiographic progression (a change in SASSS ≥2) over 2 years. There were no differences between patients with radiographic progression and those without in terms of gender, HLA-B27 positivity, disease duration, or history of clinical symptoms. Age and age at the time of diagnosis were higher for patients with radiographic progression (each p<0.001). Baseline CRP levels were higher for patients with radiographic spinal progression, but the results were equivocal (p = 0.051). Patients with radiographic spinal progression had a higher mean SASSS and a greater number of syndesmophytes at baseline (all p<0.001).

Table 2 shows the SPARCC scores for the SIJs in all 110 axSpA patients. The mean score for bone marrow edema was 9.4±12.4. The mean depth (bone marrow edema ≥ 1cm) and intensity scores were 0.6±1.4 and 1.7±3.2, respectively. The inflammatory lesion scores showed excellent interobserver reliability (ICC, 0.92–0.95). The mean SIJ SSS for fat metaplasia was 4.6±7.8, whereas those for erosion and ankyloses were 3.8±5.7 and 2.0±5.4, respectively. The SSS for fat metaplasia and ankyloses showed excellent interobserver reliability (ICC, 0.98), whereas that for erosion was very good (ICC, 0.85) and that for backfill was moderate (ICC, 0.57).

**Table 2:** Baseline sacroiliac joint MRI findings in patients with axial spondyloarthritis (n = 110).

MRI finding		Mean±SD score	N (%), score of>0	ICC (95% CI)*
Acute inflammation	Bone marrow edema	9.4±12.4	71 (65)	0.95 (0.91–0.97)
	Depth	0.6±1.4	21 (19)	0.94 (0.89–0.98)
	Intensity	1.7±3.2	30 (27)	0.92 (0.87–0.95)
Structural lesions	Fat metaplasia	4.6±7.8	51 (46)	0.98 (0.97–0.99)
	Erosion	3.8±5.7	55 (50)	0.85 (0.77–0.90)
	Backfill	0.2±0.8	8 (7)	0.57 (0.34–0.72)
	Ankylosis	2.0±5.4	18 (16)	0.98 (0.97–0.99)

MRI, magnetic resonance imaging; ICC, Interclass correlation coefficient

*ICC for each score between reader 1 and reader 2

The inflammatory lesion score for bone marrow edema and depth correlated with the ESR and CRP levels at baseline; however, this was not the case for the intensity score. None of the scores for structural lesions resulting from chronic inflammation correlated with the ESR or CRP levels (S1 Table).

Table 3 shows changes in the SASSS over 2 years in patients with or without each type of structural lesion at the time of baseline SIJ MRI. Patients with fat metaplasia or ankyloses at baseline had a significantly higher SASSS at 2 years than those without (all p<0.001). The presence/absence of erosion and backfill had no significant effect on the SASSS. When all inflammatory lesions were taken into consideration, there was no significant change in the SASSS over 2 years (S2 Table).

**Table 3:** Spinal radiographic progression over 2 years accoding to structural lesions in the sacroiliac joints observed on baseline MRI.

SIJ MRI findings at baseline	Fat metaplasia		Erosion		Backfill		Ankylosis	
	No (n = 59)	Yes (n = 51)	No (n = 55)	Yes (n = 55)	No (n = 95)	Yes (n = 15)	No (n = 89)	Yes (n = 21)
Baseline SASSS	2.5±4.5	8.6±11.7**	8.2±11.6	2.4±4.2	5.6±9.4	2.0±2.3	2.5±4.5	19.6±12.9**
Presence of syndesmophytes at baseline	0.5±1.5	2.3±3.6**	2.3±3.6	0.4±1.2**	1.4±2.9	0.3±0.7	0.5±1.3	5.7±4.2**
Change in the SASSS over 2 years	0.3±1.1	2.2±3.9**	1.7±3.6	0.6±1.9	1.3±3.0	0.4±0.7	0.8±2.7	3.0±3.1**
Change in number of syndesmophytes over 2 years	0.1±0.5	0.7±1.5*	0.6±1.4	0.2±0.6	0.4±1.1	0.1±0.4	0.2±1.3	0.8±1.6**

Data are expressed as the mean±SD

MRI, magnetic resonance imaging; SIJ, sacroiliac joints; SASSS, Stoke Ankylosing Spondylitis Spine Score

* p<0.05 and

**<0.01 (comparison between two groups (absence *vs*. presence of each MRI finding)

Univariate logistic regression analysis identified age at the time of diagnosis, HLA-B27 positivity, fat metaplasia, the presence of erosion and ankyloses on SIJ MRI, increased baseline CRP levels, and the presence of syndesmophytes at baseline as significant predictors of radiographic spinal progression at 2 years (Table 4). Treatment with sulfasalazine and TNF inhibitors showed equivocal results. Multivariate logistic analysis identified the presence of syndesmophytes at baseline as a significant predictor of spinal radiographic progression (odds ratio (OR), 5.66; 95% confidence interval (CI), 1.52–21.04). Fat metaplasia on baseline SIJ MRI was also associated with spinal radiographic progression (OR, 14.74; 95% CI, 4.71–46.14). The AUC for the model was 0.847.

**Table 4:** Univariate and multivariate analysis of factors associated with radiographic spinal progression over 2 years.

Variables	Univariate analysis		Multivariate analysis	
	OR (95% CI)	p value	OR (95% CI)	p value
**Age at time of diagnosis (years)**	1.10 (1.05–1.15)	<0.001		
**HLA-B27 positive**	0.28 (0.09–0.94)	0.040		
**Fat metaplasia score**		0.002		0.034
0	Reference		Reference	
1–5	3.12 (0.89–10.90)	0.076	2.00 (0.47–8.62)	0.348
≥6	7.66 (2.49–23.56)	<0.001	5.66 (1.52–21.04)	0.010
**Presence of erosion** *	0.38 (0.15–0.98)	0.045		
**Presence of ankyloses** *	6.42 (2.18–18.93)	0.001		
**CRP increase (>5 mg/l)**	2.59 (1.04–6.44)	0.040		
**Presence of syndesmophytes** ^**#**^	17.30 (5.88–50.87)	<0.001	14.74 (4.71–46.14)	<0.001
**Sulfasalazine treatment**	0.32 (0.10–1.00)	0.050		
**TNF inhibitor treatment**	2.48 (1.00–6.19)	0.051		

OR, odds ratio; CI, confidence interval

*Present on baseline MRI of the sacroiliac joints

#Present on baseline X-rays of the lumbar spine

In the entire cohort of patients with axial SpA (n = 110), the mean ± SD SASSS was 5.29 ± 9.12 at baseline and 6.47 ± 10.87 after 2 years (P < 0.001), resulting in a difference of 1.18 ± 2.90 units.

**Fig 1:**
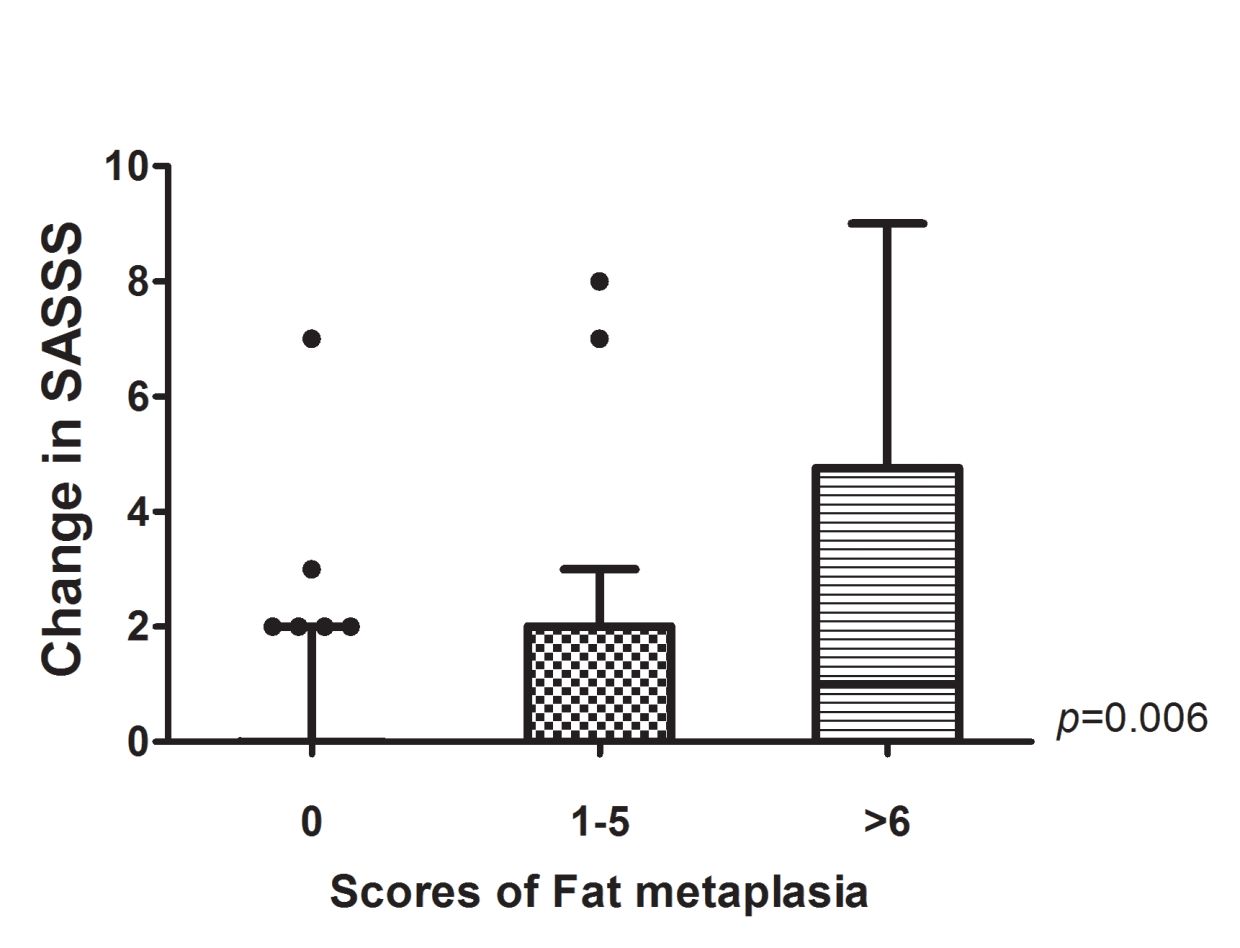
Radiographic progression according to the severity of fat metaplasia on baseline sacroiliac joint (SIJ) MRI. Spinal radiographic progression (scored according to the SASSS) was significantly greater in patients with severe fat metaplasia on baseline SIJ MRI after adjusting for baseline SASSS and TNF inhibitors treatment. P values were calculated using an analysis of covariance model, with the severity of fat metaplasia at baseline as a factor and the baseline SASSS and TNF inhibitors treatment as covariates. The bottom and top of the box were the first and third quartiles, and the band inside the box was the median. The ends of the whiskers represented 1.5 interquartiles.

Fig 1 shows the increase in SASSS for patients stratified according to the severity of fat metaplasia on baseline SIJ MRI. The patients were divided to three groups: no fat metaplasia, mild metaplasia (score 1–5), and severe metaplasia (score ≥6). The mean change in the SASSS for patients without fat metaplasia was 0.3±1.1, whereas that for patients with mild and severe fat metaplasia was 1.2±2.2 and 3.0±4.7, respectively. After adjusting for baseline SASSS and TNF inhibitor treatment, the increase in the SASSS was significantly greater for patients with severe fat metaplasia in the SIJ at baseline (p = 0.006).

## Discussion

This is the first study to examine the association between MRI findings in the SIJs and spinal radiographic progression in axSpA patients. The results show that fat metaplasia on baseline SIJ MRI and the presence of syndesmophytes at baseline are associated with an increased risk of spinal structural damage.

Several risk factors predict radiographic spinal progression in AS/axSpA. The strongest predictor of radiographic spinal progression is the presence of syndesmophytes at baseline [3,4,18]. This is supported by the results of the present study. Increased levels of acute phase reactants (e.g., the ESR or CRP) also predict spinal radiographic progression in patients with axSpA [4,19]. These parameters are markers of systemic inflammation. Smoking is also a known predictor for spinal progression [4]. Among other known predictors, the presence of structural damage at baseline, once initiate, is thought to be less dependent on inflammation [2]. It suggesting that irreversible changes in signaling associated with osteoproliferation may play an important role in spinal radiographic progression.

Focal fat infiltration, which occurs secondary to inflammation, is an early sign of repair. Fat metaplasia therefore appears to be an inherent characteristic of the tissue response in both the SIJs and spine following resolution of inflammation in SpA [20]. Here, we showed that baseline radiographic damage and fat metaplasia on SIJ MRI were independently associated with radiographic progression.

Previous MRI studies report the association between spine MRI findings and new syndesmophyte formation. However, it is debatable whether active inflammation as detected by spine MRI predicts radiographic progression. Earlier studies suggest that inflammatory lesions on spine MRI predict new syndesmophyte formation [9,21]. Recent studies suggest that syndesmophytes more likely develop at vertebral corners at which inflammation has resolved rather than at those at which inflammation persists [21,22]. However, another longitudinal study showed that fatty changes are more important than regression of inflammation [11]. Recent studies show that the majority of syndesmophytes develop in vertebral units in the absence of evidence of inflammation and/or fat metaplasia on MRI, suggesting that the relationship between inflammation detected by MRI and syndesmophyte formation is not straightforward [8,11]. Others suggest that spinal progression may be triggered, either completely or in part, independently of inflammation [23].

Here, baseline MRI findings in the SIJs suggest that inflammatory lesions are not associated with radiographic progression over 2 years. This may be because inflammatory lesions on MRI reflect transient and reversible inflammation. Neither erosion nor backfill on SIJ MRI predicted radiographic progression; however, univariate analysis revealed that fat metaplasia and ankylosis were associated with spinal progression. Fat metaplasia was an independent predictor of spinal progression after adjusting for confounders. This suggests that fat metaplasia signifies the beginning of irreversible post-inflammatory changes, as well as chronic inflammation.

The sequence of events that lead to the development of fat metaplasia in the SIJs is unclear. Little is known about the importance of this lesion, and its histopathology remains undefined. Fat metaplasia in the SIJs appears to be an inherent characteristic of the tissue response following the resolution of inflammation. Also, fat metaplasia is a key intermediary step in the development of SIJ ankylosis [20]. The results presented herein suggest that fat metaplasia in the SIJs is associated with spinal progression. The findings also indicate that fat metaplasia in the SIJs (caused by post-inflammatory changes) may affect osteoblastogenesis. The mechanisms underlying the activation of osteoproliferation, with subsequent syndesmophyte formation and ankylosis, remain unclear. One possible explanation is that fat metaplasia in the sacrum and iliac bones may influence osteoproliferation in the adjacent bone marrow. The iliac bone, sacrum, and vertebrae are the main bone marrow reservoirs, which include the stroma, bone marrow microvascular endothelial cells, adipocytes, osteoblasts, osteoclasts, and mesenchymal stem cells. Sub-chondral fat metaplasia in axSpA may affect (either directly or indirectly) osteoblastogenesis in the bone marrow. A recent *in vitro* study showed that higher triglyceride metabolism in bone tissue is associated with increased osteoblastogenesis [24]. Further studies are needed to clarify the effects of fat metaplasia in the SIJs on spinal new bone formation.

We also found that inflammatory lesions, such as bone marrow edema or depth scores, correlated with inflammatory markers, suggesting that the severity of sacroiliitis on SIJ MRI is associated with cumulative systemic inflammation. Therefore, structural lesions on SIJ MRI may reflect irreversible post-inflammatory changes. Current classification criteria for axSpA include only SIJ MRI findings, not spinal MRI findings [12]. The combination of spine and SIJ MRI did not lead to an incremental increase in value when compared with SIJ MRI alone [25]. Furthermore, the high cost and inconvenience associated with spinal MRI mean that it is not possible to scan all axSpA patients to predict spinal progression. Therefore, the improved ability of SIJ MRI to predict radiographic spinal progression in axSpA patients may be useful in daily clinical practice and may guide therapeutic decision making, particularly as previous reports suggest that continuous NSAID treatment reduces radiographic progression [26].

This study has some limitations. First, we used the SASSS system to quantify spinal radiographic progression. This system is limited in that it takes into account the structural changes in the lumbar spine, but not those in the cervical and/or thoracic spines. Second, we did not evaluate the effect of smoking on spinal progression due to insufficient baseline data. Another limitation of this study was the small sample size. Despite a trend that the patients treated with sulfasalazine or TNF inhibitors showed less radiographic progression that those who were not on these drugs, no significance was in the multivariate analysis. The lack of statistical significance may have been influenced by the low number of patients. Lastly, patients were followed for only 2 years. Since structural damage in the spine occurs slowly, longer study periods would further clarify the relevance of fat metaplasia on SIJ MRI as a predictor of spinal progression.

In conclusion, inflammatory lesions on SIJ MRI were not associated with spinal radiographic progression, whereas fat metaplasia and ankylosis were. Fat metaplasia and the presence syndesmophytes at baseline were independent predictors of spinal progression over 2 years. Further studies over longer periods are needed to confirm whether fat metaplasia on SIJ MRI is truly predictive of new bone formation in patients with axSpA.

## Supporting Information

S1 Table Correlation between findings on sacroiliac joint MRI and baseline ESR or CRP levels. (DOC)

S2 Table Changes in the SASSS over 2 years according to the type of inflammatory lesion in the saroiliac joints at baseline. (DOC)
