# Correction: Fat Metaplasia on Sacroiliac Joint Magnetic Resonance Imaging at Baseline Is Associated with Spinal Radiographic Progression in Patients with Axial Spondyloarthritis

**DOI:** 10.1371/journal.pone.0151443

**Published:** 2016-03-08

**Authors:** Kwi Young Kang, In Je Kim, Min A Yoon, Yeon Sik Hong, Sung-Hwan Park, Ji Hyeon Ju

In the Funding section, the grant number from the funder Basic Science Research Program through the National Research Foundation of Korea (NRF), funded by the Ministry of Education, Science and Technology is listed incorrectly. The correct grant number is: NRF-2014R1A1A1006695.
