# Supplementary material for: Fat Metaplasia on Sacroiliac Joint Magnetic Resonance Imaging at Baseline Is Associated with Spinal Radiographic Progression in Patients with Axial Spondyloarthritis
Source: PLoS One. 2015 Aug 13;10(8):e0135206. doi: 10.1371/journal.pone.0135206 (PMC4535979; doi:10.1371/journal.pone.0135206)
Supplement: S1 Table — (DOC) [file pone.0135206.s001.doc]

**S1 Table.** Correlation between findings on sacroiliac joint MRI and baseline ESR or CRP levels

| **MRI finding** | **ESR (mm/hr)** | |  | **CRP (mg/l)** | |
| --- | --- | --- | --- | --- | --- |
|  | *r* coefficient | p value |  | *r* coefficient | p value |
| Bone marrow edema | 0.331 | <0.001 |  | 0.343 | <0.001 |
| Depth | 0.234 | 0.014 |  | 0.202 | 0.034 |
| Intensity | 0.135 | 0.160 |  | 0.159 | 0.098 |
| Fat metaplasia | 0.089 | 0.354 |  | 0.066 | 0.493 |
| Erosion | 0.136 | 0.157 |  | 0.166 | 0.082 |
| Backfill | 0.077 | 0.423 |  | -0.020 | 0.834 |
| Ankylosis | 0.098 | 0.311 |  | 0.050 | 0.605 |
