# Supplementary material for: Fat Metaplasia on Sacroiliac Joint Magnetic Resonance Imaging at Baseline Is Associated with Spinal Radiographic Progression in Patients with Axial Spondyloarthritis
Source: PLoS One. 2015 Aug 13;10(8):e0135206. doi: 10.1371/journal.pone.0135206 (PMC4535979; doi:10.1371/journal.pone.0135206)
Supplement: S2 Table — (DOC) [file pone.0135206.s002.doc]

**S2 Table.** Changes in the SASSS over 2 years according to the type of inflammatory lesion in the saroiliac joints at baseline

|  | **Bone marrow edema** | | **Depth** | | **Intensity** | |
| --- | --- | --- | --- | --- | --- | --- |
|  | No (n=39) | Yes (n=71) | No (n=80) | Yes (n=30) | No (n=89) | Yes (n=21) |
| SASSS at baseline | 7.6±12.3 | 4.0±6.5 | 6.6±10.2 | 1.7±3.8* | 6.1±9.8 | 1.8±4.1 |
| Number of syndesmophytes at baseline | 2.1±3.6 | 0.9±2.2 | 1.7±3.2 | 0.3±1.0* | 1.6±3.0 | 0.4±1.2 |
| Change in SASSS over 2 years | 1.8±4.1 | 0.8±2.0 | 1.4±3.1±) | 0.6±2.2 | 1.2±3.0 | 0.4±1.2 |
| Change in syndesmophyte number over 2 years | 0.7±1.5 | 0.2±0.7 | 0.5±1.2 | 0.1±0.4 | 0.4±1.2 | 0.2±0.5 |

Data are expressed as the mean ± SD

SASSS, Stokes ankylosing spondylitis spine score

*p<0.05 and **p<0.01 (comparison between two groups (absence *vs.* presence of each MRI finding))
